# Arterial blood pressure monitoring in children

**DOI:** 10.1186/1824-7288-41-S2-A35

**Published:** 2015-09-30

**Authors:** Simonetta Genovesi, Ciro Corrado, Patrizia Salice

**Affiliations:** 1 Department of Health Sciences, University of Milano-Bicocca and Nephrology Unit, San Gerardo Hospital, Monza, 20900, Italy; 2 Paediatric Nephrology Unit, G. Di Cristina Children's Hospital, Palermo, 90127, Italy; 3 Centro Ipertensione, Sezione Ipertensione in Eta’ Pediatrica and Area Omogenea Malattie Cardiovascolari, IRCCS Ca’ Granda Ospedale Maggiore Policlinico, Milano, 20122, Italy

## 

In 2008 the first set of consensus recommendations for performance and interpretation of 24 hours Arterial Blood Pressure Monitoring (ABPM) in children and adolescents have been published [1]. Since then, ABPM has found increasing use in pediatrics. These recommendations have been updated in 2014 [2]. For this reason the Group of Hypertension Study of the Italian Society of Pediatrics (GISPER) has felt the need to perform an update of the Italian recommendations on this topic. The ABPM should be used by experts who know how to run it and interpret it. Proper execution is in fact necessary and only trained staff can guarantee it. Children and parents should be educated on the significance of the examination and care should be taken in selection of the appropriate size cuff according to the size of the child's arm. For the interpretation of the ABPM data, the age- and sex-specific percentiles of Wühl et al [3] are the preferred reference nomograms. Table 1 shows the suggesting schema for interpretation of ABPM values, in defining Blood Pressure categories. On the contrary that in the adult, the ABPM in children cannot be considered the gold standard for the diagnosis of high blood pressure, which must be done by measuring office Blood Pressure values, according to the criteria established by the National High Blood Pressure Education Program Working Group on High Blood Pressure in Children and Adolescents [4]. Other important differences compared to adults are using the pressure load (defined as pathological in the presence of a number of measurements of systolic or diastolic Blood Pressure values >25% of total) to define the different blood pressure categories and the presence of Pre-Hypertension among Blood Pressure categories. The ABPM also allows to identify individuals with White Coat or Masked Hypertension, clinical situations that, in children as in adults, suggest the need for careful follow-up. It was shown that both of these conditions can be associated with the presence of early organ damage, such as left ventricular hypertrophy in children. Finally, ABPM can give important information about Blood Pressure variability, distinguishing subjects with normal nocturnal Blood Pressure dip (>10% compared to the day, dipping), from non-dipping children.

**Table 1:** Suggesting schema for Ambulatory Blood Pressure levels interpretation in children (modified by ref.2)

CLASSIFICATION	OFFICE BLOOD PRESSURE	AMBULATORY SYSTOLIC OR DIASTOLIC BLOOD PRESSURE	SYSTOLIC OR DIASTOLIC LOAD
Normal Blood Pressure	<90th percentile	<95th percentile	<25%

White Coat Hypertension	>95th percentile	<95th percentile	<25%

Pre-Hypertension	>90th percentile or >120/80 mmHg	<95th percentile	>25%

Masked Hypertension	<95th percentile	>95th percentile	>25%

Ambulatory Hypertension	>95th percentile	>95th percentile	25-50%

Severe ambulatory Hypertension	<95th percentile	>95th percentile	>50%
